# Oral Health Changes in Patients with Oral and Oropharyngeal Cancer

**DOI:** 10.1016/S1808-8694(15)30609-1

**Published:** 2015-10-18

**Authors:** Caio Perrella de Rezende, Marcelo Barboza Ramos, Carlos Henrique Daguíla, Rogério Aparecido Dedivitis, Abrão Rapoport

**Affiliations:** 1 MSc on Health Sciences at Hospital Heliópolis, HOSPHEL, São Paulo, Specialist in periodontics.; 2 Specialist in dental implants, MSc student on Health Sciences at Hospital Heliópolis, HOSPHEL, São Paulo.; 3 Specialist in dental implants, MSc student on Health Sciences at Hospital Heliópolis, HOSPHEL, São Paulo.; 4 PhD in medicine at the ENT and Head and Neck Surgery Graduate School at UNIFESP - Escola Paulista de Medicina, MD.; 5 Associate professor at the Department of Surgery at the São Paulo University Medical School, Coordinator of the graduate program on Health Sciences at Hospital Heliópolis, HOSPHEL, São Paulo. Stomatology Service and Head and Neck Surgery Service at Complexo Hospitalar Heliópolis, HOSPHEL, São Paulo, and civil society organization ‘Young Dentists Association’, São Bernardo do Campo.

**Keywords:** oral hygiene, oral neoplasms, dmf index, periodontal index

## Abstract

Inflammatory disease and oral trauma are relevant factors for patients with oral cancer.

**Aim:**

This paper aims to assess the association between oral hygiene, periodontal disease, oropharyngeal and oral cancer.

**Materials And Method:**

In this cross-sectional prospective study, fifty subjects with untreated oral and oropharyngeal squamous cell carcinoma were compared to fifty cancer-free subjects, paired by age and gender. They answered an oral health questionnaire and underwent oral examination to assess periodontal disease and dental health, as per the CPITN. Periodontal disease classification and CPITN assignment were done according to WHO guidelines.

**Results:**

Periodontal examination and the CPITN elicited the differences between the two groups, with evidences of advanced disease among the subjects with oral or oropharyngeal cancer, confirmed by the presence of periodontal pockets with depths of 6mm or greater in 76% of the subjects evaluated, while only 10% of the subjects in the control group showed the same level of disease. No relevant differences were observed in the DMF index and oral hygiene between both groups.

**Conclusion:**

The findings indicate that there is an association between cancer and more severe periodontal disease regardless of oral hygiene and dental health status.

## INTRODUCTION

The two main risk factors for oral cancer are smoking and alcohol abuse. There is a synergistic effect among these factors and a direct relationship between the level of abuse and exposure to these substances. However, other factors have also been associated with oral and oropharyngeal cancer, among which are biologic agents such as the human papillomavirus (HPV), precarious oral hygiene, previous history of respiratory or digestive tract tumor and excessive exposure to ultraviolet light (lip cancer). Most oral cancer cases are diagnosed at later stages of the disease. Self-examination and periodic visits to a trained professional are recommended for earlier diagnosis^1^, ^2^, ^3^.

Brazilian guidelines recommend the promotion of oral hygiene and regular visits to dental care professionals as preventive measures. Careful clinical examination of the mouth must be performed in all visits to the medical doctor, even if the complaints are not located there. High risk individuals (smokers and drinkers) should have their mouths systematically examined, and those with suspicious lesions must be immediately referred to a specialized reference center so that the proper diagnostic procedures can be performed^4^.

As very little progress has been observed in early diagnosis, the best approach is to reduce exposure to risk factors. Therefore, it is important to identify inflammatory disease and oral trauma in the population in general and in oral cancer patients^5^, ^6^, ^7^, ^8^.

Oral health has not been consensually defined in the literature. One may consider that a patient presents inadequate oral health when inflammatory processes, infection, or trauma set in or poor hygiene is administered^9^. Trauma may be caused by poorly fitted prosthetics or fractured teeth^10^; lesions are predominantly found in the tongue, in the gingival jugal region, and on the palate^11^.

The lack of definitive studies describing the inflammatory and trauma-related aspects in the populations affected or not by oral cancer calls for a study whose purpose is to determine the possible correlations between oral health status and the predisposing factors for oral and oropharyngeal squamous cell carcinoma.

## MATERIALS AND METHOD

This is a cross-sectional prospective study that included patients seen between February of 2003 and January of 2004. The disease-free control group was recruited within the same time span, and participants were selected and paired by gender, age, and social-economic status. Patients were not paired for smoking or drinking, nutritional status, clinical staging, or lesion sub-sites. This research project was approved by the Ethics Research Committee or our institution under permit 253 on September 9, 2003.

All clinical information was obtained through interviews that looked at issues connected to past and present oral hygiene habits of the participants. Associated disease data and all relevant cancer-related clinical aspects were gathered from the patients’ medical records. Oral clinical examination was conducted to look for signs of periodontal disease and tooth decay.

Patients wearing dental prosthetics were asked to remove them before oral examination to allow for ample visualization of existing trauma in usually covered areas. Those in whom trauma was found were sent to assessment and treatment. Individuals in the control group presenting suspicious lesions were excluded from the study and sent to specialized evaluation.

The principles defined by the World Health Organization (WHO) in the form of the Community Periodontal Index of Treatment Needs (CPITN) were used to detect periodontal disease. This index assesses the depth of periodontal pockets in a scale from 1 to 4. Dental health status was determined by the DTMF, defined by the WHO to look at dental caries, lost, and capped teeth9.

At the end of the examination each participant was assigned a score to account for their dental health status. The higher the score, the worse the clinical condition in terms of lost and capped teeth and caries

Student's T-test was used to statistically compare the age averages of both groups, while non-parametric chi-square test was adopted to draw the differences involving oral health variables. For all tests a threshold of 5% was used to determine statistical significance.

## RESULTS

**Chart 1:**
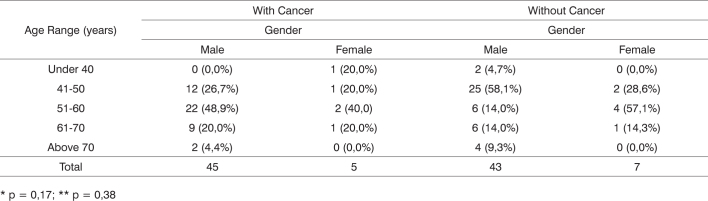
Group gender and age distributions.

Chart 1 presents the distribution of individuals with and without cancer in relation to gender and age. No statistically significant differences were found between the groups, as all participants were older than 40 years of age.

* p = 0,17; ** p = 0,38

**Chart 2:**
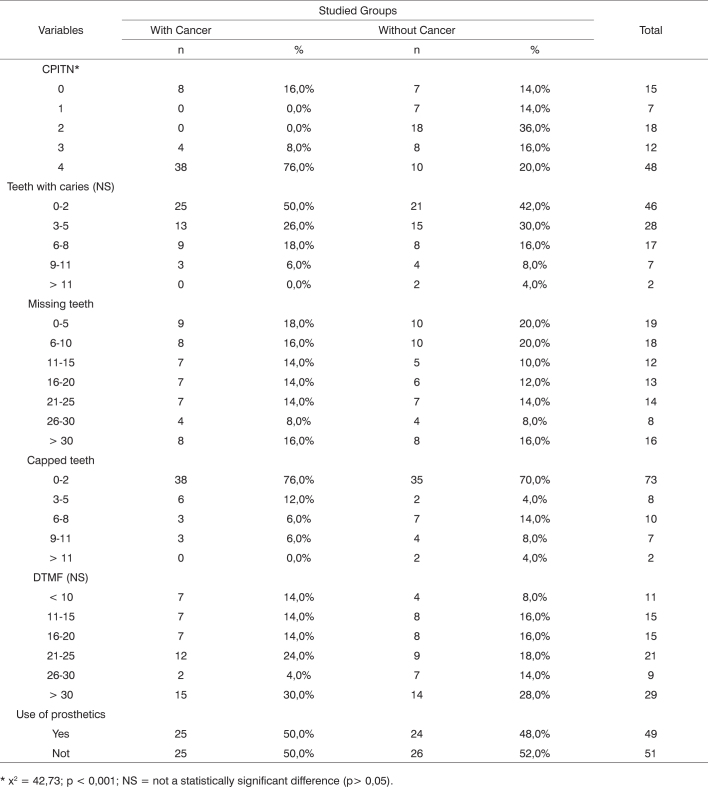
Oral examination findings.

Chart 2 describes the results gathered from the oral examination results from both groups. Periodontal examination and the CPITN show a significant difference among cancer patients, as 76% of them had advanced periodontal disease and periodontal pockets deeper than 6 millimeters. In contrast, only 10% of the control group members had similar degrees of disease.

* x^2^ = 42,73; p < 0,001; NS = not a statistically significant difference (p> 0,05).

No significant differences were found for the number of lost and capped teeth or teeth with caries, with similar findings in the clinical examination of dental specimens. Use of prosthetics was also similar for both groups.

Most individuals (100% of cancer patients and 92% of the control group) brush their teeth up to twice a day, while none of them, regardless of the group they belonged to, used mouthwash, dental floss or other oral hygiene accessories.

## DISCUSSION

Epidemiological research has shown that smoking and drinking are the most important risk factors for oral cancer^10^. However, aspects such as poor oral health status, poorly fitted prosthetics, income level, eating habits, and periodontal status may also play an adjuvant role in the development of oral and oropharyngeal cancer^12^. In a study looking at patients with chronic kidney disease undergoing hemodialysis it was found that age, diabetes, smoking, and albumin levels were independently associated with the degree of periodontitis^13^.

The association between smoking, drinking, and poor oral health significantly increases the risk of cancer. Risk of oral cancer among males with these three factors is 7.7 times greater than that of individuals without any such risk factors^14^.

Various factors such as personal habits, professional activity, and place of residence when combined with smoking, drinking, and poor oral hygiene may lead to increased prevalence rates of oral cancer^15^.

In a case-control study on oral cancer, tooth loss and other aspects related to dental care were similar among participants, but oral health status as indicated by gum bleeding, dental calculi, and mucosal irritation was worse among cancer patients when compared to control group participants^16^.

The results presented in this study indicate similar levels of dental health status among the groups, as no significant difference was found in the DTMF. Another interesting finding is that both observed groups cared very little for their oral hygiene, as none of the participants regularly used dental floss, mouthwash, and brushed their teeth fewer than three times a day, as seen in other studies^12^.

Cell lesion caused by poor dental health, trauma due to partial or complete tooth malformation, chipped or broken teeth and lack of hygiene may be associated with the course of changes that lead to the onset of oral cancer^17^. However, the relationship between disease and dental variables still could not be clearly proven, as other determining factors such as drinking, smoking, eating habits, income level, and culture are at play^18^.

Some of the issues pertaining to oral health such as injury caused by poorly fitted prosthetics and poor oral hygiene may be associated with cancer in the upper airways^17^. The use of dental prosthetics is not a risk factor in itself^19^.

According to the results observed, the status of periodontal tissues was worse in cancer patients when compared to control individuals. A case-control study enrolling 100 patients with upper airway squamous cell carcinoma and 214 without evident disease showed that oral hygiene and oral health status were worse among cancer patients, who also brushed their teeth less often and paid fewer visits to dental care professionals than control individuals. Calculi and periodontal pockets of 3mm or more were found in 40.9% of cancer patients and in 22% of the control group^20^, as also found in our study.

The stratified squamous epithelium that covers the oral cavity of older participants changes and becomes thinner, less elastic, slowly moving into a state of atrophy. Local immune response changes, increasing the risk of trauma and infection. The oral mucosa becomes a site for common inflammatory ulcerative diseases, infection, and tumor^21^.

Periodontal disease is caused by chronic infection induced by inflammatory responses and leads to the collapse of teeth supporting tissues. Maintaining the equilibrium between the host's defense system and the microorganisms in the gingival sulcus is key to health preservation. Cell death and proliferation are important phenomena that regulate these activities, and disturbances in this system are often connected to diseases as cancer, AIDS and rheumatoid arthritis^22^.

We could not establish significant causal relationship between daily use of mouthwash and oral cancer^23^. The study group was analyzed for the possibility of increased risk among mouthwash users, as ethanol is one of the ingredients found in some commercially available products^24^. No alterations were observed, as almost none of the participants used mouthwash.

The best way to fight oral cancer is prevention, through early diagnosis and elimination of risk factors. The failure of drinking and smoking cessation programs brings about the need to assess other secondary factors. Health education programs and initiatives that promote periodic oral examination are possibly the best tools to help reduce the high prevalence rates of oral cancer in our community^25^.

## CONCLUSION

The results indicate the existence of a correlation between periodontal disease and oral and oropharyngeal cancer. Cancer patients had more severe periodontal disease, and no correlation was established between oral and oropharyngeal cancer and oral hygiene and dental health. Longitudinal studies considering other variables such as staging, nutritional status, and habits should be conducted so as to make this association more evident, determining more clearly the role periodontal disease has as a risk factor for cancer.
